# Dr. Carey W. Howe

**Published:** 1909-08

**Authors:** 


Dr. Carey W. Howe, of Buffalo, died at his home in this city early in June. His wife died only a day or two later, and Dr. Howe's brother, Darius B. Howe, died next day after Mrs. Howe's death. Thus we record three deaths in one family within four days.

